# COVID-19 and Transplant Patients: Challenges, Risks, and Evolving Strategies

**DOI:** 10.3390/vaccines13030318

**Published:** 2025-03-17

**Authors:** Mariarosaria Campise

**Affiliations:** Department of Nephrology, Dialysis and Kidney Transplantation, Fondazione IRCCS Ca’ Granda Ospedale Maggiore Policlinico, 20122 Milan, Italy; mariarosaria.campise@gmail.com

The first cases of COVID-19 were reported in December 2019 in Wuhan, China. The disease, caused by the SARS-CoV-2 virus, is believed to have originated from a zoonotic spillover, possibly linked to a market selling live animals in damp conditions. The exact source remains a topic of scientific debate, with theories ranging from natural transmission from bats to potential leaks from a laboratory [1,2].

Initially, local authorities in Wuhan attempted to manage the outbreak without raising international alarm. However, by late January 2020, the virus had spread beyond China’s borders, reaching countries such as Thailand, Republic of Korea, Japan, and USA, as well as European nations. The World Health Organization (WHO) declared COVID-19 a Public Health Emergency of International Concern on 30 January 2020. Just over a month later, on 11 March 2020, the WHO officially declared COVID-19 a pandemic, recognizing its rapid global spread and severe consequences [3,4].

Governments around the world responded to COVID-19 with varying degrees of urgency. Aggressive testing, contact tracing, and quarantine measures, were implemented to contain early outbreaks. However, misinformation and healthcare infrastructure limitations soon became a cause for concern. Lockdowns, travel restrictions, and social distancing measures were widely imposed. These measures, though necessary, had severe economic and social consequences. Businesses shut down, unemployment rates soared, and educational institutions moved online, disrupting millions of students’ learning experiences. The psychological impact of prolonged isolation also became evident, leading to a rise in mental health issues worldwide.

The scientific community mobilized rapidly to understand the virus and develop countermeasures.

Early treatments focused on managing symptoms and preventing severe complications.

The COVID-19 pandemic posed significant challenges to the global healthcare system, especially in vulnerable populations such as solid organ transplant (SOT) recipients. Due to their immunosuppressed status, these patients are at an increased risk for severe SARS-CoV-2 infections, leading to higher associated complications and mortality rates compared to the general population. In general, the mortality rate was higher in all SOT patients: those undergoing kidney [5,6], lung [7], liver, and combined transplants [8]. In SOT patients, the treatment of COVID-19 requires a delicate balance between controlling viral replication and maintaining adequate immunosuppression. Indeed, immunosuppression, necessary to prevent organ rejection was identified as a contributing factor to the higher susceptibility and severity of infections in this group. Furthermore, comorbidities common among SOT patients, such as diabetes, hypertension, and chronic kidney disease, further increased the risk of severe COVID-19 outcomes.

Several therapies were attempted including Chloroquine/Hydroxychloroquine, Azithromycin, lopinavir-ritonavir, Ivermectin, corticosteroids, convalescent plasma, heparin, zinc, Montelukast, and monoclonal antibody. However, these drugs including antivirals, have shown highly variable effectiveness or yielded mixed results [9].

A breakthrough in COVID-19 treatment came with the development of vaccines.

By December 2020, less than a year after the pandemic began, the first COVID-19 vaccines—Pfizer-BioNTech and Moderna—received emergency use authorization in several countries. This was a historic achievement in vaccine development. Soon after, other vaccines, including AstraZeneca, Johnson & Johnson, and Sinopharm, followed, providing hope for mass immunization and pandemic control [10,11].

However, the efficacy of vaccines in SOT recipients has been a subject of concern because immunosuppressive therapy may sensibly reduce the immune response to vaccines. Their efficacy in preventing infection and severe disease may be lower than in the general population, but the paramount advantage of vaccinations is their ability to reduce the severity of infection [12].

To enhance vaccine-induced protection, many studies have explored the administration of additional doses. Research published in the *Lancet* demonstrated that a third dose of an mRNA COVID-19 vaccine significantly improved the immune response in SOT recipients who had previously exhibited suboptimal responses to the standard two-dose regimen [13].

Just as vaccines began rolling out, new variants of SARS-CoV-2 emerged. The Alpha, Beta, Delta, and Omicron variants demonstrated the virus’s ability to mutate, sometimes increasing transmissibility and reducing vaccine effectiveness. These variants led to new waves of infections and hospitalizations, particularly in regions with low vaccination rates [14,15].

Misinformation and vaccine hesitancy also hindered efforts to reach herd immunity. Anti-vaccine movements, fueled by distrust in pharmaceutical companies and governments, led to resistance against vaccination campaigns, prolonging the pandemic’s impact [16].

Among other concerns, the pandemic highlighted deep social inequalities. Low-income communities and marginalized groups suffered disproportionately due to limited access to healthcare, job losses, and overcrowded living conditions that increased infection risks. Women, particularly those in caregiving roles, have been forced to leave work since schools and daycare centers closed. Furthermore, some countries could not afford the high cost of vaccines set by the manufacturers, especially low-income countries in Africa. To overcome this problem, COVID-19 Vaccines Global Access (COVAX) was created [17]. By 31 December 2023, COVAX had delivered 2 billion doses of COVID-19 vaccines to 58 lower-income economies, preventing over 2 million deaths and achieving 57% of two-doses coverage compared to the global average of 67% [18].

The delivery of vaccines will continue into 2025 in those countries that requested further doses in 2024.

On 5 May 2023, the World Health Organization officially declared the end of a pandemic that began just over three years earlier, on 11 March 2020.

The COVID-19 pandemic has been a challenge; however, it also accelerated advancements in technology and healthcare. Telemedicine became a mainstream option for medical consultations, reducing the strain on hospitals [19]. Remote work and online learning platforms saw widespread adoption, changing traditional work and education models.

Artificial intelligence played a crucial role in pandemic management, from predicting outbreaks to accelerating drug discovery. Digital health tools, such as contact tracing apps and vaccination passports, became essential in monitoring and controlling the spread of the virus [20].

Today, COVID-19 has been downgraded to the causative agent of a respiratory tract infection. However, for some patients, such as SOT recipients, it can develop into a high-risk infection if it is not early diagnosed and treated. The lessons learned from all the lives lost must not be forgotten.

Investing in pandemic preparedness, promoting scientific literacy, and fostering international cooperation are essential to ensuring better future health resources for humanity. The legacy of COVID-19 should be one of resilience, innovation, and a commitment to a healthier, more equitable world.
